# Uncertainty quantification in neural-network based pain intensity estimation

**DOI:** 10.1371/journal.pone.0307970

**Published:** 2024-08-01

**Authors:** Burcu Ozek, Zhenyuan Lu, Srinivasan Radhakrishnan, Sagar Kamarthi

**Affiliations:** Mechanical and Industrial Engineering Department, Northeastern University, Boston, Massachusetts, United States of America; University of Lagos Faculty of Engineering, NIGERIA

## Abstract

Improper pain management leads to severe physical or mental consequences, including suffering, a negative impact on quality of life, and an increased risk of opioid dependency. Assessing the presence and severity of pain is imperative to prevent such outcomes and determine the appropriate intervention. However, the evaluation of pain intensity is a challenging task because different individuals experience pain differently. To overcome this, many researchers in the field have employed machine learning models to evaluate pain intensity objectively using physiological signals. However, these efforts have primarily focused on pain point estimation, disregarding inherent uncertainty and variability in the data and model. A point estimate, which provides only partial information, is not sufficient for sound clinical decision-making. This study proposes a neural network-based method for objective pain interval estimation, and quantification of uncertainty. Our approach, which enables objective pain intensity estimation with desired confidence probabilities, affords clinicians a better understanding of a person’s pain intensity. We explored three distinct algorithms: the bootstrap method, lower and upper bound estimation (*Loss*_*L*_*)* optimized by genetic algorithm, and modified lower and upper bound estimation (*Loss*_*S*_) optimized by gradient descent algorithm. Our empirical results demonstrate that *Loss*_*S*_ outperforms the other two by providing narrower prediction intervals. For 50%, 75%, 85%, and 95% prediction interval coverage probability, *Loss*_*S*_ provides average interval widths that are 22.4%, 7.9%, 16.7%, and 9.1% narrower than those of *Loss*_*L*_, and 19.3%, 21.1%, 23.6%, and 26.9% narrower than those of bootstrap. As *Loss*_*S*_ outperforms, we assessed its performance in three different model-building approaches: (1) a generalized approach using a single model for the entire population, (2) a personalized approach with separate models for each individual, and (3) a hybrid approach with models for clusters of individuals. Results demonstrate that the hybrid model-building approach provides the best performance.

## 1. Introduction

Failing to treat pain properly can have severe consequences. Untreated pain can significantly impact the quality of life and cause physical or mental suffering. Incorrectly managed pain can result in over-prescription or under-prescription. While over-prescription can lead to opioid dependency and drug-seeking behavior, under-prescription can cause avoidable suffering [1, 2]. The key to preventing untreated or inappropriately managed pain and deciding on the required intervention is to assess its presence and severity [3]. Clinicians employ various pain assessment methods, such as the Visual Analog Scale (VAS), in which a patient expresses their pain level on a 10 cm line, indicating the absence of pain with 0 and the most severe pain imaginable with 10. Similarly, in the Verbal Rating Scale (VRS), patients use descriptive terms ranging from “none” to “excruciating,” and in the Numeric Rating Scale (NRS), patients pick a number between 0 and 10 to express their pain intensity [4]. Clinicians use these pain scales for accurate and efficient pain measurement, aiding in early diagnosis, disease monitoring, and evaluating therapeutic efficacy [5].

Even though the VAS, NRS, or VRS methods are easy to deploy in clinical settings, assessing pain intensity accurately is challenging because pain is subjective and depends on individual physiological, emotional, cognitive, and communication differences [6]. For instance, one person may find a specific level of pain mildly irritating, while another may find the same pain excruciating. Moreover, there are limitations in verbally expressing pain levels in certain patient groups, such as non-verbal children and in medical conditions like sedation and paralysis [7]. These limitations can lead to poor pain management and potential harm. Automated and objective pain intensity assessment has gained popularity among machine learning researchers. Machine learning models can learn from data by automatically detecting and using patterns to predict pain intensity or derive new insights [8].

In the literature, many researchers have studied machine learning models such as logistic regression, decision trees, support vector machines, and neural networks to assess pain intensity objectively. These algorithms learn from facial expressions, body movements, electrodermal activity, electrocardiogram, and electromyogram collected from individuals [1, 9, 10]. This data, however, is subject to noise and uncertainty due to factors such as patient motion or muscle artifacts, natural environmental conditions like temperature fluctuations and background noise, changes in skin resistance due to sweating or hydration levels, and individual body differences [11, 12]. Additionally, machine learning models encounter challenges due to factors such as inherent randomness, hyperparameter settings, model assumptions, and complexity [13, 14]. These challenges cause an inevitable uncertainty [15, 16]. An iterative proportional-integral interval estimation provides linear discrete-time systems with stability and precision in the presence of uncertainties in system states and disturbances [17]. Similarly, iterative interval estimation-based fault detection offers a robust technique for discrete-time T–S fuzzy systems, underscoring the importance of capturing and managing uncertainties [18].

At present, objective pain intensity assessment research focuses only on point estimation, disregarding the variability in the data, uncertainty in the model, or both [19, 20]. Point estimations tend to produce overconfident predictions. Overconfident incorrect predictions can be harmful in clinical settings. Understanding the level of uncertainty in pain intensity predictions is critical. It is possible to achieve this by capturing inherent uncertainty in the model inputs and parameters and quantifying the uncertainty in the model response [20].

Generally, Neural Network (NN)-based Prediction Interval (PI) methods effectively quantify uncertainty [19, 21]. A PI is an interval estimate for an (unknown) target value. In contrast to a point estimate, a PI includes the lower and the upper bounds within which the actual target value is likely to fall with a desired probability [21–23].

The two metrics assess the quality of the PIs: (1) *accuracy*, representing Prediction Interval Coverage Probability (PICP), and (2) *dimension*, quantifying Prediction Interval Width (PIW). In literature, researchers employ four traditional methods, namely delta, Bayesian, bootstrap, and mean-variance estimation-based to create NN-based PIs [22, 24]. These methods, however, demand high computational resources or strong assumptions about the data or the model. Additionally, their primary goal is to maximize PICP, but not to minimize PIW. A 100% PICP can be obtained by setting a large value for the upper bound and a small value for the lower bound of PIs. However, this approach provides no helpful information about the target value. In practice, to achieve high-quality PIs require maximizing PICP and at the same time minimizing PIW [21, 25].

In this work, we explore pain intensity interval estimation by implementing three distinct NN-based models: bootstrap method, lower and upper bound estimation model (*Loss*_*L*_) optimized by genetic algorithm, and modified lower and upper bound estimation model (*Loss*_*S*_) optimized by gradient descent algorithm. Our findings reveal that the *Loss*_*S*_ consistently outperforms the other two modeling approaches by providing narrower pain intensity intervals. We then conduct a comprehensive analysis of the applications of the *Loss*_*S*_ approach across three distinct model-building approaches: (1) a generalized approach where only one model is built for the overall population, (2) a personalized approach where separate models are built tailoring for each individual, and (3) a hybrid approach where separate models are created for clusters of individuals. To our knowledge, this study is the initial effort to develop a prediction interval method for uncertainty quantification in the field of pain intensity estimation.

We organized the rest of the paper as follows. The background section explores the objective pain assessment concept and prediction interval framework. The methods section introduces the data exploration and PI construction process. The results and discussion section presents three NN-based PI estimation methods and three model-building approaches. It identifies the best performing method and model-building approach. The conclusion section highlights the results and limitations of this work and provides insights into the prospective trajectory of the work.

## 2. Related work

This section consists of two subsections. Section 2.1 reviews recent studies in automated pain assessment that have concentrated on leveraging biomarkers and machine learning models for point estimation. Section 2.2 discusses prediction interval techniques to accurately capture and represent the inherent uncertainty in the models and data.

### 2.1 Pain assessment

Automated and objective pain assessment has gained increasing interest among many machine learning researchers over the years [26]. Physiological signals, such as brain activity, cardiovascular activity, and electrodermal activity, have emerged as a promising source of information for developing objective pain assessment methods. These signals are connected to the autonomic nervous system and play an essential role in pain response [15, 27]. Pain researchers have widely used physiological signals to develop objective and automated pain assessment methods [1, 9, 10, 28–33].

One of the most well-known datasets of pain-related physiological signals is the BioVid Heat Pain dataset created by Walter et al. [34]. This dataset consists of electrodermal activity (EDA), electrocardiogram (ECG), electromyogram (EMG), and electroencephalography (EEG). EDA measures the skin’s electrical properties (skin conductance) [11, 35]. ECG analyzes the electrical activity related to the heart [36]. EMG is the measurement of muscle activity [27]. EEG measures the brain’s electrical activity [37]. Researchers report that EDA is one of the most valuable signals for automated and objective pain assessment [1, 11, 33, 38–43]. Several researchers used EDA signals from BioVid dataset in the literature to develop different machine learning models with low Root Mean Squared Error (RMSE) [1, 33, 43].

Kächele et al. [43] employed a random forest algorithm on EDA signals from the BioVid dataset; they reported the best performance RMSE as 1.01. Martinez et al. [33] applied linear regression, support vector regression, neural network, fully-connected recurrent neural networks, and long short-term memory networks to the EDA signals. They achieved RMSE of 1.36, 1.37, 1.32, 1.29, and 1.29, respectively. Pouromran et al. [1] explored many machine learning models which gave low RMSE: linear regression (RMSE: 1.18), SVR (RMSE: 1.15), neural networks (RMSE: 1.15), random forest (RMSE: 1.15), KNN (RMSE: 1.17), and XGBoost (RMSE: 1.13) on BioVid EDA signals. **Table 1** presents an overview of studies that develop machine learning models utilizing EDA for point estimations of pain intensity, considering it a continuous variable.

**Table 1:** Overview of studies that developed machine learning models utilizing EDA for point estimations of pain intensity.

Study	Machine Learning Model	RMSE
Kächele et al., [43]	Random Forest	1.01
Martinez et al., [33]	Linear Regression	1.36
	Support Vector Regression	1.37
	Neural Network	1.32
	Fully-Connected Recurrent Neural Networks	1.29
	Long Short-Term Memory Networks	1.29
Pouromran et al., [1]	Linear Regression	1.18
	Support Vector Regression	1.15
	Neural Networks	1.15
	Random Forest	1.15
	K-Nearest Neighbors	1.17
	XGBoost	1.13

These studies developed point estimation algorithms that do not consider the uncertainty in the data and the model. Including uncertainty in making robust diagnosis and treatment decisions in clinical settings is crucial. Neglecting uncertainty may result in either over or under-treatment. Over-treatment and over-prescription of opioids can increase the risk of addiction and overdose. Conversely, under-treatment can deteriorate mental and physical health, reduce the quality of life, extend hospital stays, and cause patient dissatisfaction [44, 45].

### 2.2 Prediction interval framework

Prediction, which plays a crucial role in decision-making, is highly susceptible to any source of uncertainty affected by input data, measurement errors, model approximation errors, parameter uncertainty, and model bias [46]. Causes of uncertainty in the prediction framework are grouped into model uncertainty (epistemic uncertainty) and irreducible variance (data noise variance or aleatoric uncertainty) [22]. The uncertainty framework in prediction is formulated as follows:

$$\sigma_{y}^{2}=\sigma_{model}^{2}+\sigma_{noise}^{2}$$ (1)

When predicting, the impact of uncertainty term $\sigma_{y}^{2}$ should not be overlooked. Researchers have commonly used NN-based PIs to identify and analyze uncertainty. A PI includes the estimated upper and lower bounds between which the unknown future value of the target *y* = *f*(*x*) is expected to lie, with a specified confidence level, (1−*α*)% [47, 48]. In literature, researchers have used two criteria to evaluate the quality of a PI: Prediction Interval Coverage Probability (PICP) and Prediction Interval Width (PIW). PICP is the probability that the estimated PI will cover the actual target value [49, 50]. A high PICP can easily be achieved by accepting a wide PI, but it has no predictive value. Hence, consideration of Mean Prediction Interval Width (MPIW), the average difference between the estimated lower and upper bounds, is necessary for creating a high-quality PI [22].

A narrow PI (i.e., small MPIW) with high PICP is the most desirable outcome in practice. Thus, it is valid to say that there is a tradeoff between these two criteria (MPIW and PICP) when evaluating the quality of a PI. A higher desirable PICP could lead to a wider MPIW and vice versa [51]. Researchers have proposed several methods in the literature for constructing NN-based PI estimation models that address the tradeoff between PICP and MPIW. In literature, there are four traditional methods to build NN-based PI estimation models: (1) bootstrap, (2) delta, (3) mean-variance estimation (MVE), and (4) Bayesian [24]. However, they all face common disadvantages: they demand high computational resources and make strong assumptions about the model or input data.

Khosravi et al. [19] developed a new approach, the Lower Upper Bound Estimation (LUBE), to overcome the limitations of the aforementioned traditional NN-based PI estimation methods. In LUBE, the NN model has two output neurons, one for the upper bound and the other for the lower bound of the PIs. The parameters of the NN model (biases and weights) are optimized considering a novel loss function. This loss function does not directly minimize the regression error; instead, it aims to improve MPIW and PICP simultaneously. Khosravi et al. [19] employed a simulated annealing (SA) method to optimize this novel loss function, considering it nonlinear, complex, discontinuous, and non-differentiable. LUBE is more reliable than traditional techniques and requires no assumptions about the data or model distributions.

Since the LUBE method performs well and does not impose restrictions on the data distribution and model structure, many researchers adopted it by utilizing various evolutionary optimization algorithms. Quan et al. [52] optimized LUBE using particle swarm optimization (PSO) for electrical load forecasting. Lian et al. [47] adopted the LUBE method to generate NN-based PIs for the landslide displacement; they combined particle swarm optimization and gravitational search algorithm (GSA) to optimize a neural network. Shen et al. [53] developed a multi-objective artificial bee colony algorithm (MOABC) incorporating multi-objective evolutionary knowledge (EKMOABC), and optimized a wavelet neural network to create PIs with the LUBE for wind power forecasting.

Although LUBE performs well, researchers reported some limitations. When PIs are zero, the loss function finds its global minimum at zero [22]. The loss function is highly nonlinear, complex, discontinuous, and non-differentiable, and hence, only evolutionary algorithms, such as simulated annealing and particle swarm optimization, could optimize this loss function. These algorithms require a slow training process and the standard neural network training techniques like the gradient descent algorithm (GD) struggle to effectively optimize this particular loss function [47].

To overcome these challenges, researchers improved to the LUBE function and modified the loss function or treated it as a multi-objective optimization problem [54–60]. Quan et al. [52] modified the loss function by improving the interval width assessment metric. They modified the LUBE loss function’s PIW based on the mean absolute percentage error and trained NN using the new metric called PI normalized root-mean-square width, which penalizes model’s large error terms. Secondly, they used an additional loss function to consistently assess the quality of PIs using the scoring rule called SCORE developed by Winkler et al. [61], which rewards a narrow PI and penalizes when the target value is not within the PI. Lian et al. [47] proposed a single hidden layer feedforward NN with random hidden weights. This means that in addition to the output weights, the input weights and hidden biases of the NN are randomly chosen. This randomization of the input weights and hidden biases introduces more variability into the model, potentially allowing it to capture a broader range of patterns and relationships in the data. In addition, the optimization of the output weights is performed using a hybrid evolutionary algorithm called PSOGSA (Particle Swarm Optimization and Gravitational Search Algorithm). This hybrid approach combines the strengths of both algorithms to more effectively optimize the output weights and improve the model’s overall performance. Ak et al. [21] and Shen et al. [53] approached this problem as a multi-objective optimization problem. Ak et al. [21] utilized a multi-objective genetic algorithm, the non-dominated sorting genetic algorithm–II (NSGA-II), to construct PIs. NSGA-II solves optimization problems where multiple conflicting objectives must be optimized simultaneously (PICP and PIW). NSGA-II maintains a population of candidate solutions (often called individuals or chromosomes) and evolves them over successive generations to find a set of Pareto-optimal solutions. Shen et al. [53] introduced the EKMOABC technique to train the network. The EKMOABC algorithm optimizes the construction of prediction intervals by iteratively adjusting parameters based on a multi-objective function that minimizes interval width while maximizing coverage probabilities.

Pearce et al. [22] introduced modifications to tackle convergence towards a global minimum when PIW is zero and to ensure the differentiability and compatibility with GD. They replaced the step function of PICP (1 when the actual target value is inside the PI, 0 otherwise) with a differentiable approximation by incorporating a softening factor and sigmoid function. Next, they replaced the conventional PIW definition with a captured PIW approach. This change involves calculating PIW only for data points where the actual value falls within the upper and lower bounds of the PI. The sole consideration of the captured data mitigated the potential influence of non-captured data on the calculation of PIW. Furthermore, they modified the impact of PIW in the loss function by transforming multiplicative terms into additive terms. Similarly, replacing the exponential term with the squared term altered the effect of the PICP. Lastly, they included additional hyperparameters to provide a high confidence in PICP (see Section 3 for details).

In this study, we present an NN-based PI method that integrates two distinct loss functions, Loss_L_ and Loss_S_, which are discussed in detail in the next section. Subsequently, we compare the PIs generated by these loss functions with those constructed using the bootstrap method, which we consider as the baseline for the evaluation. The first loss function, Loss_L_, explicitly targets the challenges associated with traditional NN-based methods, which often require significant computational resources and rely on strong assumptions about the model or input data for uncertainty quantification in pain intensity estimation. To address the limitation of the Loss_L_ function, namely the convergence towards incorrect optimal solutions in specific scenarios, we adopted the second loss function, Loss_S_. By exploring Loss_L_ and Loss_S_ along with the comparative bootstrap method, we aim to address these challenges and improve the accuracy and robustness of pain intensity estimation within the uncertainty quantification framework.

## 3. Methods

### 3.1 BioVid Heat Pain database and feature extraction

In this work, we train the models using the BioVid Heat Pain database, a publicly available dataset, to construct PIs for pain intensity estimation [34]. This database includes (1) physiological modalities (EDA, EEG, ECG, and EMG), and (2) behavioral modalities (facial expression) of 87 participants. The pain intensity is the target variable (label) in this dataset; it varies from 0 to 4. In the BioVid experiments, each participant was exposed to personalized four different temperature levels (T1, T2, T3, and T4). The absence of external temperature stimuli was considered the control temperature level (T0). In the current work, a regression model estimates the continuous numeric pain intensity.

This study uses the EDA signal, widely accepted as a neurocognitive stress indicator in pain recognition research [1, 11, 35, 62]. We extract features from the EDA using the "Canonical Time-series Characteristics" defined by Lubba et al. [63]. These features consist of basic statistical measures of time-series data, stationarity, entropy, linear correlations, physical nonlinear time-series analysis techniques, linear and nonlinear model parameters, predictive power, and fits [63]. In this work, we use the 22 most informative features (see **Table 2**) identified by Lubba et al. [63]. We use all these 22 features because our exploration demonstrates that models constructed with these features consistently outperform those created with different subsets of feature combinations.

**Table 2:** The top 22 most informative features extracted from the EDA signal.

Time-Series Feature Category	Description
Distribution	Mode of z-scored distribution with a 5-bin histogram
	Mode of z-scored distribution with a 10-bin histogram
Simple temporal statistics	The longest period of consecutive values above the mean
	Time intervals between successive extreme events above the mean
	Time intervals between successive extreme events below the mean
Linear autocorrelation	First 1/e crossing of the autocorrelation function
	First minimum of the autocorrelation function
	Total power in the lowest fifth of frequencies in the Fourier power spectrum
	Centroid of the Fourier power spectrum
	Mean error from a rolling 3-sample mean forecasting
Nonlinear autocorrelation	Time-reversibility statistic, ⟨(x_t+1_ − x_t_)^3^⟩_t_
	Auto mutual information, m = 2,τ = 5
	First minimum of the auto-mutual information function
Successive differences	The proportion of successive differences exceeding 0.04σ (Mietus et al. [64])
	The longest period of successive incremental decreases
	Shannon entropy of two successive letters in equiprobable 3-letter symbolization
	Change in correlation length after iterative differencing
	Exponential fit to successive distances in 2-d embedding space
Fluctuation Analysis	The proportion of slower timescale fluctuations that scale with DFA (50% sampling)
	The proportion of slower timescale fluctuations that scale with linearly rescaled range fits
Others	Trace of covariance of transition matrix between symbols in the 3-letter alphabet
	Periodicity measure (Wang et al. [65])

After conducting data cleaning procedures, such as missing value analysis, the dataset contained 8612 observations, each with 22 features. These features are standardized via min-max normalization. The label assigned to each instance corresponds to the level of pain intensity, which varies between 0 and 4.

This study assesses the merit of prediction intervals (PIs) generated by three distinct model-building approaches. First, we create a "generalized" model common to all subjects covered in the study. Second, we develop 87 "personalized" models, one for each subject. Third, we group the 87 subjects into 4 clusters, and then construct one dedicated model for each cluster of subjects. We employ the k-means the *k*-means clustering technique to group the individuals based on their EDA signal feature vectors. For each subject, we construct 110-dimensional vectors (22 features x 5 pain intensity levels) using the average of the normalized features in each pain level. The *k*-means algorithm is an iterative clustering technique employed to partition a dataset into *’k* = 4*’* distinct clusters. Initially, 4 centroids are randomly initialized within the data space. Subsequently, each data point is assigned to the nearest centroid based on the Euclidean distance metric. Through iterative updates, wherein centroids are recalculated as the mean of the data points within each cluster, the algorithm converges to a solution, resulting in clusters with minimum intra-cluster distance and maximum inter-cluster distance [66].

Cluster-specific models are built for each cluster; this approach is a "hybrid" of the generalized and personalized approaches [1]. All models are trained using 90% of the observations and evaluated with the remaining 10% with a ten-fold cross-validation scheme.

### 3.2 Development of prediction intervals by neural network

Section 3.2.1 delves into the neural network structure; Section 3.2.2 introduces the evaluation metrics for evaluating the quality of PIs; Section 3.2.3 explains the NN-Based PIs constructed by the bootstrap method; Sections 3.2.4 and 3.2.5 provide detailed information about the loss functions employed to optimize the prediction intervals.

#### 3.2.1 Neural network structure

Our study employs an NN-based PI model to assess the uncertainty of predictions. The network consists of one input layer with 22 neurons each representing a distinct EDA feature. The output layer has two neurons: one for the lower bound and the other for the upper bound of the PI. The network has two hidden layers, whose size was selected between 10 to 120 neurons depending on the scenario.

The hidden layer utilizes the Rectified Linear Unit (ReLU) function as an activation function, while the output layer employs a linear function as an activation function. The neural network architecture, including the number of hidden layers, the number of neurons in each hidden layer, and the choice of activation functions, is optimized through the hyperparameter tuning process. **Table 3** illustrates the parameter search space.

**Table 3:** The neural network parameters are optimized through a search space hyperparameter tuning process.

Parameter	Search space
# of Hidden Layers	[1,4]
# of Hidden Neurons in a Layer	[10,150]
Activation Function for Hidden Layers’ Neurons	[ReLU, Hyperbolic Tangent, Linear]

#### 3.2.2 PI assessment

We evaluate the quality of PIs by employing PICP and MPIW measures. We aim to create PIs as narrow as possible (i.e., PIs with small MPIW) with a PICP as high as possible. We calculate PICP as:

$$PICP=\frac{1}{n}\sum_{i=1}^{n}k_{i}$$ (2)

where *n* is the number of observations,

$$k_{i}=\left\{ \begin{array}{l} 1,\;if\;L(X_{i})\leq X_{i}\leq U(X_{i})\\ 0,\;else \end{array}\right.$$ (3)

where, *L*(*X*_*i*_) is the lower bound and *U*(*X*_*i*_) is the upper bound of the PI of the *i*^*th*^ observation.

We then calculate MPIW as:

$$MPIW=\frac{1}{n}\sum_{i=1}^{n}U(X_{i})-L(X_{i})$$ (4)

We calculate normalized mean prediction interval width (NMPIW) as follows:

$$NMPIW=\frac{MPIW}{R}$$ (5)

where *R* represents the range of the target, *R* = max(*y*)−min(*y*), and NMPIW represents the width of the prediction interval relative to the target range.

#### 3.2.3 Bootstrap method

We utilize the bootstrap method, one of the most employed techniques, to construct PIs. It involves building a specific number *(B)* of NN models by resampling the training data from the original data with replacement. The outputs of the NN models are averaged to estimate the actual regression mean. The output of the NN also calculates the variance of predictions. The resulting mean and variance are used to construct the PIs. This method has the following drawbacks: (1) it is computationally expensive when dealing with large datasets, and (2) it could provide inaccurate estimations due to bias when the observation set is small or not representative [22, 67].

#### 3.2.4 *Loss*_*L*_

We employ the loss function from LUBE [19] to evaluate the BioVid Heat Pain database. In this work, we refer to LUBE’s loss function as Loss_L_, which is calculated by the following:

$${Loss}_{L}=\frac{MPIW}{R}(1+\gamma (PICP)e^{-\eta (PICP-\mu )})$$ (6)

where *R* represents the range of the target variable, which, in this application, is the pain intensity measured on a 0–4 scale; *μ* and *η* are constant hyperparameters; *μ* which represents the confidence level associated with PIs, which can be set to 1−*α*; *η* amplifies any small discrepancy between PICP and *μ*. The term *γ*(*PCIP*) is a step function that evaluates the quality of PIs on the test set. For training, *γ*(*PCIP*) is considered as 1 [19], where

$$\gamma =\left\{ \begin{array}{l} 0,\;PICP\geq \mu \\ 1,\;PCIP<\mu \end{array}\right.$$ (7)

**Fig 1:**
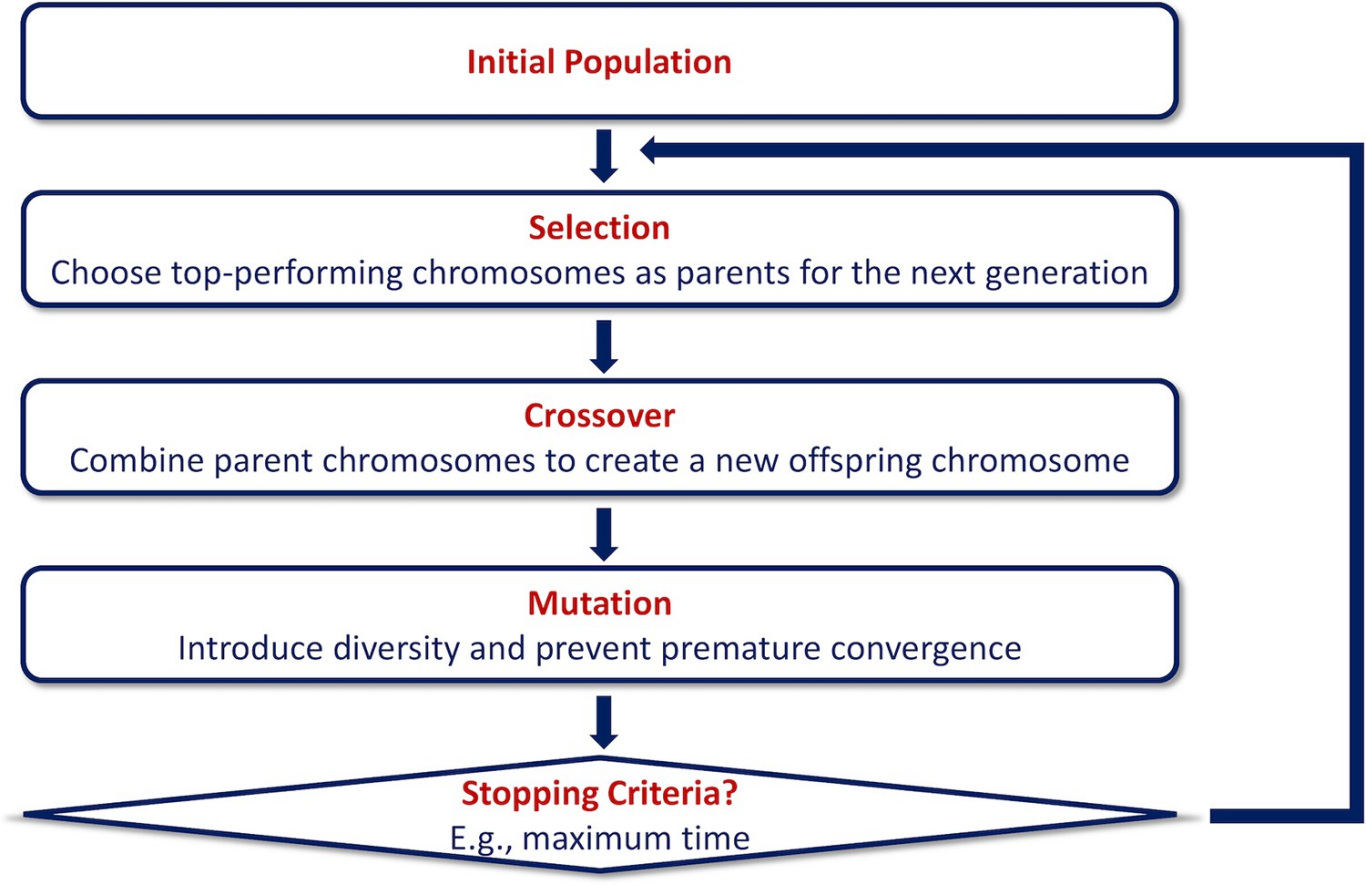
GA consists of selection, crossover, and mutation steps.

We train the NN model with a Genetic Algorithm (GA), a search heuristic inspired by the natural evolution theory [68]. It repeatedly changes initial solutions by choosing individuals from the current population as parents and uses them to build the children for the next generation at each stage. The algorithm can find the optimal solution as the population evolves with each iteration [69, 70]. We implement the genetic algorithm (GA) using the PyGAD Python Library, developed by Gad, an open-source Python library explicitly designed for constructing genetic algorithms and optimizing machine learning algorithms [71]. This implementation is carried out within the PyTorch framework. **Fig 1** shows the GA process.

**Table 4** shows the parameters of GA and Loss_L_ and their respective search space for hyperparameter tuning. The optimal values of the parameters are found by hyperparameter tuning.

**Table 4:** GA and loss function (Loss_L_) parameters are optimized using their search spaces through the hyperparameter tuning.

Parameter	Search space
# of Solutions (GA parameter)	[10,20]
# of Parent Mating (GA parameter)	[5,10]
% Genes (GA parameter)	[10,20]
*η* (loss function parameter)	[25,100]
*μ* (loss function parameter)	[0.5,0.95]

#### 3.2.5 *Loss*_*S*_

We implement a modified version of the LUBE’s loss function [22] and explore its performance on the BioVid dataset. In the modified version, the formulas for MPIW and PICP are changed. We refer to the modified LUBE’s loss function as the soft loss function, denoted as Loss_S_. We use MPIW_S_ and PICP_S_ to represent the modified versions of MPIW and PICP, respectively.

The soft loss function *Loss*_*S*_ is calculated by:

$${Loss}_{S}={MPIW}_{S}+\lambda \frac{\eta }{\alpha (1-\alpha )}max{\left(0,(1-\alpha )-({PICP}_{S}\right)}^{2}$$ (8)

where,

$${PICP}_{s}=\frac{1}{n}\sum_{i=1}^{n}\sigma (s(X_{i}-L(X_{i}))⊗\sigma (s(U(X_{i})-X_{i})$$ (9)

$${MPIW}_{S}=\frac{1}{\sum_{j=1}^{n}k_{j}}\sum_{i=1}^{n}k_{i}[U(X_{i})-L(X_{i})]$$ (10)

In the above expressions, *σ* is the sigmoid function; ⊗ is the matrix multiplication operator; *s* is the softening factor *λ* is a Lagrangian to control the impact of MPIWs and PICP_S_; η is a constant hyperparameter that represents the batch size; *μ* = 1−*α* represents the confidence level associated with PIs; MPIW_S_ captures MPIW only when the condition, *L*(*X*_*i*_) ≤ y_*i*_ ≤ *U*(*X*_*i*_), is true; PICP_S_ is calculated by replacing the step function, *k*_*i*_, with a smooth sigmoid function.

Loss_S_ is differentiable and compatible with gradient descent (GD) training; GD is an iterative optimization algorithm that identifies the local minimum of a function. The algorithm calculates the gradient of the objective function and adjusts the model parameters in the opposite direction to the gradient [72]. The detailed steps of the GD method are as follows:

1. Start by randomly initializing the parameters for the model, i.e., NN parameters.
2. Compute the loss function (*Loss*_*S*_).
3. Compute the gradient of the loss function with respect to the parameters, which corresponds to the first-order derivative of the function at the local point (slope at the local point).
4. Take a step in the opposite direction to the gradient and move towards the minimum of the loss function. This step is taken by multiplying the gradient by a scalar value called the learning rate (ξ) and subtracting the results from the current parameter values.
$$\theta =\theta -\xi \frac{\partial J(\theta )}{\partial \theta }$$ (11)
  where θ is the current NN parameter values (weights); ξ is the learning rate; J(θ) is the loss function calculated by the θ; the partial derivative, $\frac{\partial J(\theta )}{\partial \theta }$, is the gradient of the loss function with respect to the current parameter values.
5. Update parameters with the new values obtained in the previous step.
6. Repeat steps 2–5 until the gradient becomes close to zero or a stopping criterion is met; a gradient close to zero indicates that the parameters have converged to a minimum of the loss function.

For the application of gradient descent, we utilize the Adaptive Moment Estimation (Adam) optimizer from the Keras library.

The optimal values of the parameters for Loss_S_ and the GD algorithm are found by hyperparameter tuning. **Table 5** shows the parameter search space.

**Table 5:** GD and soft loss function (Loss_S_) parameters are optimized through the hyperparameter tuning process within their search spaces.

Parameter	Search space
Learning rate (GD parameter)	[0.001, 0.1]
Decaying rate (GD parameter)	[ 0.000001, 0.0001]
*λ* (loss function parameter)	[5,30]
η (loss function parameter)	[35,240]
μ = 1 –a (loss function parameter)	[0.5,0.95]
*S* (loss function parameter)	[10,220]

## 4. Results and discussion

Section 4.1 compares the performance of the PIs of the generalized models built using the bootstrap method (baseline model), Loss_S_ optimized by GD, and Loss_L_ optimized by GA. Section 4.2 analyzes the relationship between MPIW and PICP in the hyperparameter tuning process. Section 4.3 discusses the performance of PIs constructed using Loss_S_ optimized by GD for the generalized, personalized, and hybrid models.

### 4.1 A comparative analysis: NN-based PIs versus bootstrap, Loss_L_ by GA, and Loss_S_ by GD

This section provides a comparative analysis of PIs generated by Loss_S_, Loss_L_, and bootstrap. The NN-based models are trained using the EDA signals of all 87 subjects. The goal is to construct PIs with the maximum coverage probability and minimum width. We use 22 features extracted from the EDA signals and the pain intensity level as the continuous response variable between 0 to 4.

**Fig 2:**
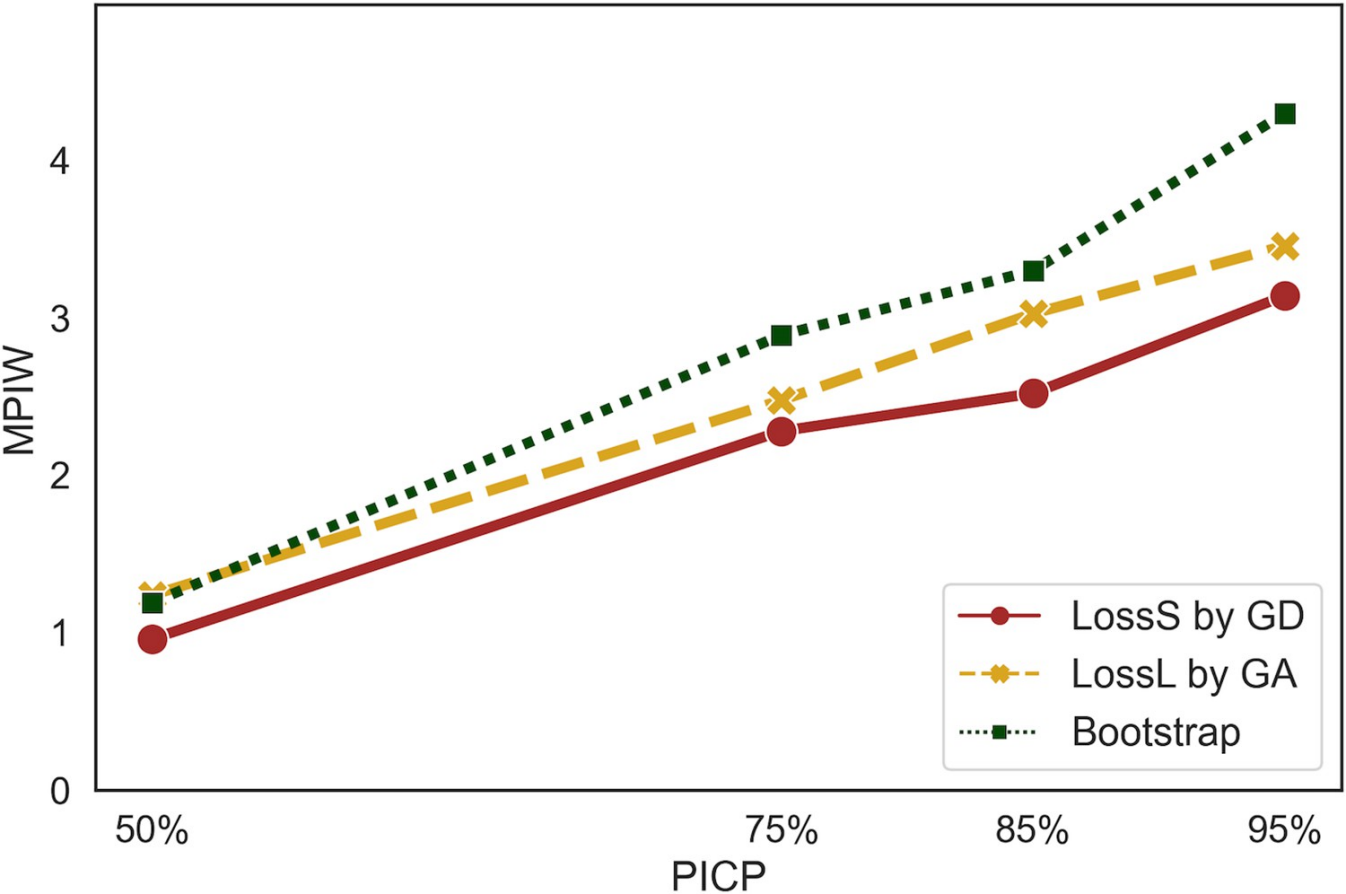
Performance comparison of Loss_S_ by GD, Loss_L_ by GA, and bootstrap methods; Loss_S_ outperforms by yielding a narrower PIW across all PICP values.

**Fig 2** illustrates the PICP and MPIW values for bootstrap, Loss_L_ by GA, and Loss_S_ by GD methods. The findings demonstrate that Loss_S_ outperforms the others. Specifically, for PICP values of 50%, 75%, 85%, and 95%, Loss_S_ gives MPIWs that are 22.4%, 7.9%, 16.7%, 9.1% narrower than the results of Loss_L_ respectively, and 19.3%, 21.1%, 23.6%, 26.9% narrower than the results of the bootstrap method.

For example, when considering a coverage probability of 75%, Loss_S_ yields a PIW of 2.3, whereas Loss_L_ produces a PIW of 2.5, and the bootstrap method constructs a PIW of 2.9. Similarly, at a coverage probability of 85%, Loss_S_ generates a PIW of 2.5, Loss_L_ constructs a PIW of 3.0, and the bootstrap constructs a PIW of 3.3.

The results presented in **Fig 2** indicate the existence of a direct connection between PICP and MPIW. For example, in the Loss_S_ method, with a coverage probability of 85%, the MPIW is approximately 2.52. This result means that on average, when the pain intensity level is 3 on a 0–4 scale, the estimated range, with an 85% coverage probability, typically spans from 1.31 to 3.51. When aiming for a higher coverage probability (95%), the MPIW increases accordingly. For instance, a pain intensity level of 3 on a 0–4 scale results in a PI of 0.59 and 3.89 on average.

### 4.2 Analysis of the relationship between MPIW and PICP

**Fig 3:**
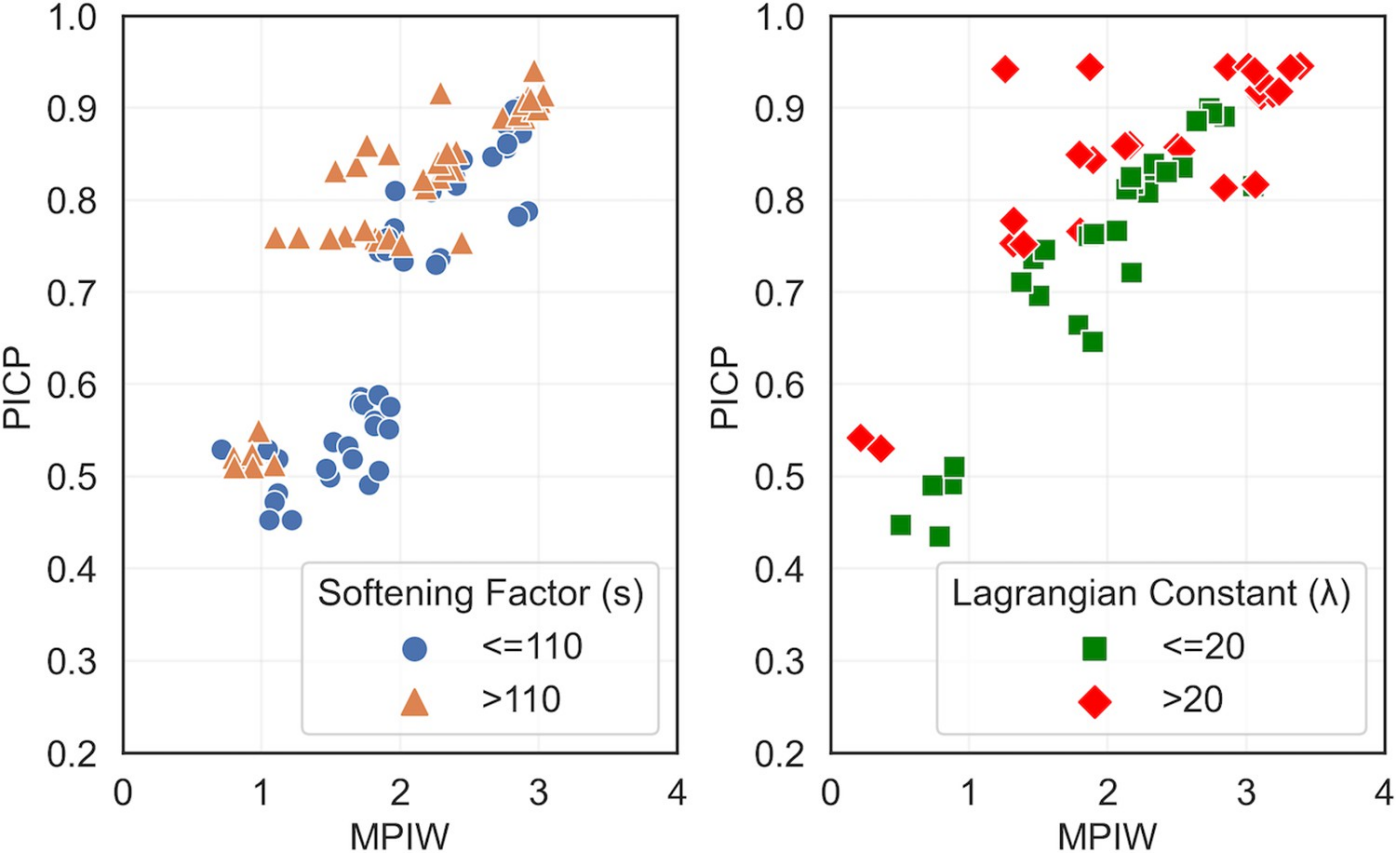
(a) higher softening factor (s) value improves the PICP. (b) A higher Lagrangian multiplier (λ) value results in a slightly higher PICP.

For a good understanding of the relation between MPIW and PICP, we first need to study the impact of Loss_S_ function hyperparameters on the training process. In **Fig 3**, we observe how the hyperparameters of Loss_S_, λ (the Lagrangian constant that determines the relative importance of MPIW_S_ and PICP_S_) and *s* (the softening factor, which relaxes the original PICP definition) affect the MPIW_S_ and PICP_S_. **Fig 3A** focuses on the effect of *s* while keeping all other parameters constant; **Fig 3B** examines the impact of λ while keeping all other parameters constant. When we compare different *s* values at the same MPIW_S_ level, we see higher *s* values generally result in better PICP_S_ values. This means an increase in *s* value yields higher coverage probability with a narrower PIW. For example, for an MPIW_S_ range between 1.5 and 2, *s* values smaller than 110 result in a PICP_S_ between 50% and 60%, but with *s* values larger than 110, we can achieve PICP_S_ higher than 75%, most of the time. **Fig 3B** shows an increasing trend, indicating that higher λ values result in slightly higher PICPs.

### 4.3 NN-based PI estimation with Loss_S_ by GD algorithm

The NN-based PI model trained with the Loss_S_ and optimized by the GD algorithm outperforms the Loss_L_ optimized by the GA and bootstrap methods for the generalized model. Therefore, we investigate the application of the NN-based PI model trained with the Loss_S_ and optimized by the GD algorithm application on the two other model-building approaches: personalized model and hybrid model. This section provides more detailed information about each of these model-building approaches.

#### 4.3.1 Generalized model

We use the EDA signals of all 87 subjects to develop generalized models that are applicable to the entire population. **Table 6** presents the results of the generalized models for various coverage probabilities:50%, 75%, 85%, and 95%. **Table 7** presents the mean of the upper and lower bounds for each pain level as PICP varies.

**Table 6:** The generalized model results demonstrate how MPIW and NMPIW change as PICP varies.

PICP	MPIW	NMPIW
50%	0.96	0.24
75%	2.28	0.57
85%	2.52	0.63
95%	3.14	0.79

**Table 7:** The mean of the upper and lower bounds for each pain level in the generalized model as PICP varies.

	GENERALIZED MODEL							
	50% PICP		75% PICP		85% PICP		95% PICP	
Target	Lower Bound	Upper Bound	Lower Bound	Upper Bound	Lower Bound	Upper Bound	Lower Bound	Upper Bound
0	1.32	2.09	0.17	2.47	0.21	3.12	0.06	3.28
1	1.48	2.32	0.31	2.9	0.51	3.37	0.16	3.45
2	1.68	2.75	0.56	2.99	0.74	3.47	0.31	3.5
3	2.15	3.27	1.13	3.35	1.31	3.51	0.59	3.68
4	2.83	3.77	2.23	3.82	2.1	3.84	1.12	3.89

For instance, when considering a 75% coverage probability, the MPIW measures approximately 2.28. This outcome signifies that, on average, for a pain intensity level of 3 on 0–4 scale, the estimated range typically extends from 1.13 to 3.35. While the constructed PIs exhibit reasonable performance, the key strength lies in the generalizability of the proposed approach. With this method, as new patients arrive, their pain intensity prediction intervals can be constructed without requiring additional model training, which has significant implications for clinicians who seek to objectively evaluate the pain intensity levels of their patients instead of relying solely on self-reported information for pain treatment and management.

#### 4.3.2 Personalized model

We develop personalized models, custom trained for each subject personal data. In this approach, 87 different personalized models are created, one for each of the 87 subjects. **Table 8** shows the averaged PICP, MPIW, and NMPIW values across 87 personalized models. **Table 9** presents the mean upper and lower bounds for each pain level derived from the averages of the personalized models.

**Table 8:** Compared to the generalized model results, the PI widths of the personalized model are wider.

PICP	MPIW	NMPIW
50%	1.63	0.41
75%	2.44	0.61
85%	2.89	0.72
95%	3.12	0.78

**Table 9:** The mean upper and lower bounds for each pain level averaged across participants for various PICP values.

	PERSONALIZED MODEL							
	50% PICP		75% PICP		85% PICP		95% PICP	
Target	Lower Bound	Upper Bound	Lower Bound	Upper Bound	Lower Bound	Upper Bound	Lower Bound	Upper Bound
0	0.05	2.04	0.28	2.75	0.04	2.96	0.01	3.2
1	0.6	2.4	0.38	2.93	0.21	3.17	0.07	3.42
2	0.5	2.51	0.63	3.15	0.34	3.36	0.41	3.66
3	1.58	2.7	0.99	3.48	0.71	3.61	0.8	4
4	2.01	3.03	1.49	3.72	1.42	4.04	1.2	4.19

In comparison with the generalized model’s findings, the PIWs are larger for personalized models. This is mainly because personalized models have a very limited number of observations for training, making it difficult for the models to learn. In addition to the poor performance, the lack of generalizability makes the personalized models unsuitable for clinical settings. In the case of a new patient arriving at the hospital with no prior patient history, a new model must be developed and trained on the patient-specific EDA observations, which may not be feasible in a clinical setting. Nonetheless, this approach can help build smart, personalized devices that can collect vast amounts of data from individuals and use this personalized data to train and customize models for each individual.

#### 4.3.3 Hybrid model

In a clinical setting, personalized models are neither generalizable nor practical. A machine learning model trained with the population data may not yield accurate predictions for individuals who significantly differ in physiological characteristics. Therefore, we create a hybrid of generalized and personalized models to estimate PIs. With this aim, we use a clustering-based approach to group patients based on their EDA features. Here, the subjects with similar EDA features are clustered, and NN-based PIs are constructed for each cluster separately. In this method, upon the arrival of a new patient, we place the patient in the nearest cluster based on EDA signals and subsequently utilize the cluster-specific model to construct PIs. **Table 10** displays the number of individuals belonging to clusters and the PICP, MPIW, and NMPIW values of the PIs for each cluster. **Table 11** presents PIs, including the average upper and lower bounds across pain levels, which are calculated for PICP values of 50%, 75%, 85%, and 95%.

**Table 10:** The hybrid model results include PICP, MPIW, and NMPIW.

Cluster	Number of Individuals	PICP	MPIW	NMPIW
1	27	50%	0.37	0.09
		75%	1.47	0.37
		85%	1.85	0.46
		95%	2.50	0.63
2	24	50%	0.42	0.11
		75%	1.38	0.35
		85%	1.89	0.47
		95%	2.38	0.60
3	20	50%	0.59	0.15
		75%	1.77	0.44
		85%	1.69	0.42
		95%	2.81	0.70
4	16	50%	0.42	0.11
		75%	1.48	0.37
		85%	2.04	0.51
		95%	2.67	0.67

**Table 11:** The mean upper and lower bounds for each pain level averaged across clusters for PICP values of 50%, 75%, 85%, and 95%.

	HYBRID MODELS							
	50% PICP		75% PICP		85% PICP		95% PICP	
Target	Lower Bound	Upper Bound	Lower Bound	Upper Bound	Lower Bound	Upper Bound	Lower Bound	Upper Bound
0	0.94	1.72	0.72	2.25	0.09	2.49	0.16	2.56
1	1.34	2.23	0.85	2.48	0.65	2.95	0.25	2.96
2	1.89	2.58	1.15	2.69	1.2	3.175	0.36	3.22
3	2.4	2.8	1.45	3.13	1.75	3.48	0.715	3.46
4	2.98	3.4	1.95	3.545	2.73	3.83	1.54	3.71

The number of subjects in clusters 1 through 4 is 27, 24, 20, and 16, respectively. Compared to generalized and personalized models, the cluster-specific models perform better. The average MPIWs for clusters are 0.44, 1.52, 1.86, and 2.5 for 50%,75%,85%, and 95% PICP, respectively.

**Fig 4:**
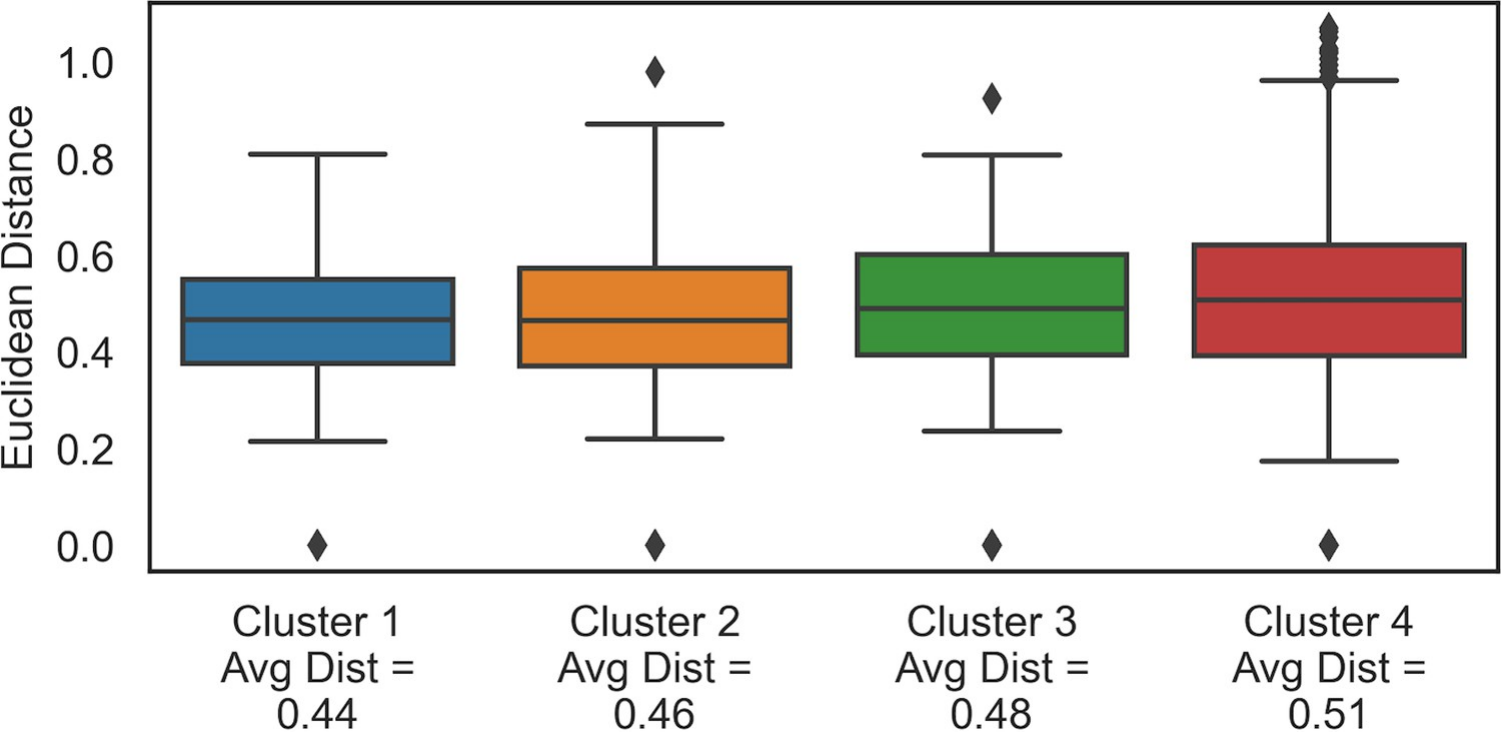
The distribution of pairwise Euclidean distances for Clusters 1, 2, 3, and 4.

The pairwise Euclidean distance between the subjects in each cluster is calculated, and the distribution of distances is plotted in **Fig 4**. The average inter-subject distances for clusters 1 through 4 are 0.44, 0.46, 0.48, and 0.51, respectively. The average inter-subject distances of clusters 1 and 2 are smaller than those of clusters 3 and 4. Clusters with smaller average inter-subject distances yield slightly better quality PIs. However, overall, all these clusters perform similarly. **Fig 4** demonstrates that Cluster 4 has more outliers than the other clusters.

**Fig 5:**
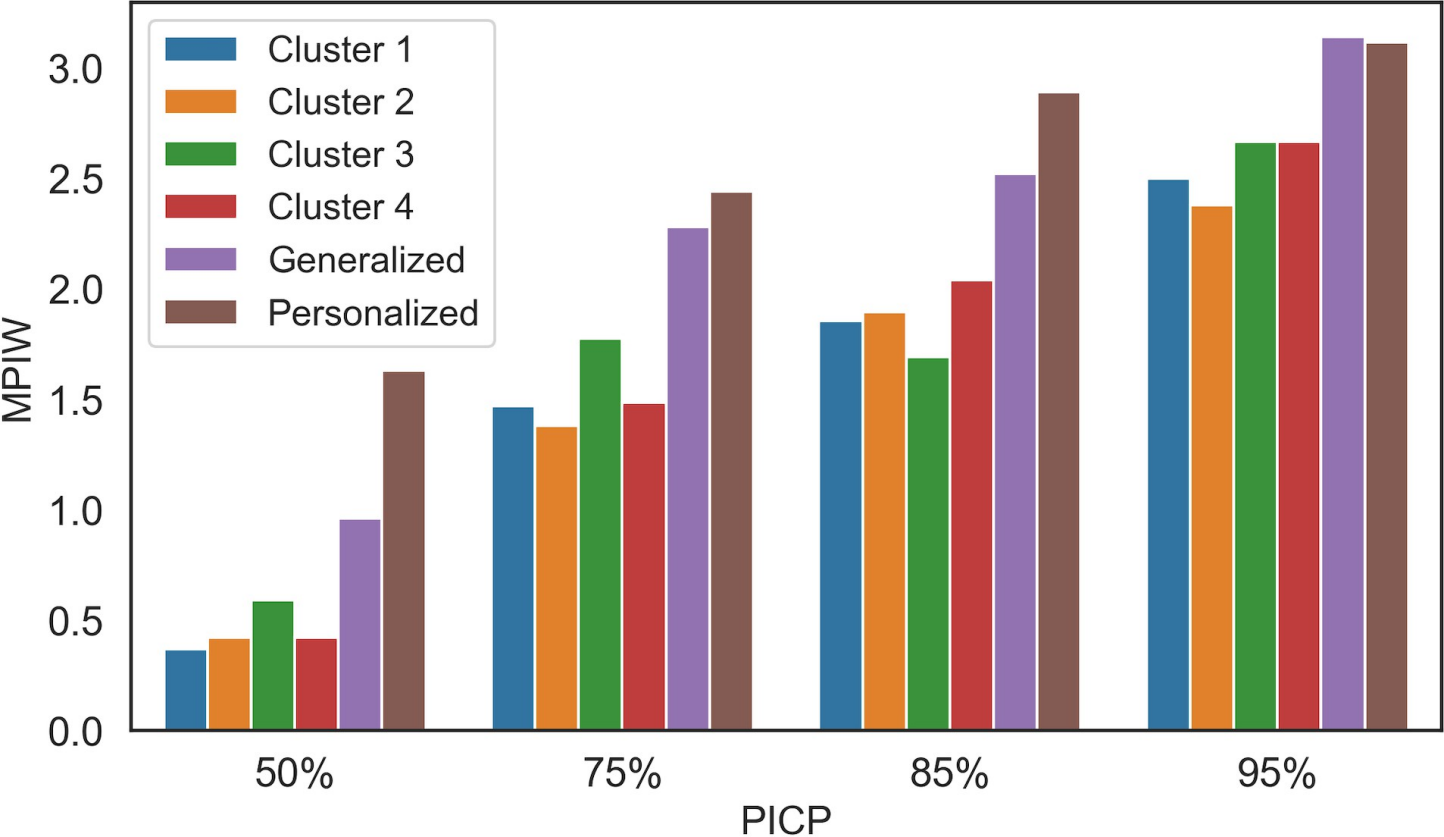
The hybrid approach, which utilizes a clustering technique, outperforms the other models and is considered a viable option for implementation in clinical settings.

**Fig 5** illustrates the values of MPIW_S_ and PICP_S_ for each approach. The first four bars in the figure represent the interval width values of each cluster, followed by the fifth bar for the generalized model and the last for the personalized model. These bars are presented for the PICP_S_ values of 50%, 75%, 85%, and 95%, respectively.

According to the results, using a clustering-based approach can yield significantly better quality PIs for pain intensity. Identifying subgroups of individuals who exhibit similar EDA patterns can enhance the quality and efficiency of constructing PIs. Such an approach is found to be the most effective among various models and has practical applications in clinical settings.

## 5. Conclusion

In this work, we develop an NN-based prediction interval method to estimate pain intensity while capturing the prediction uncertainty. We use EDA signals from the BioVid Heat Pain database of 87 individuals to develop and assess our models. We extract 22 features from the EDA signals, including basic statistics of time-series values, stationarity, fits, entropy, physical nonlinear time-series analysis techniques, linear and nonlinear model parameters, linear correlations, and predictive power. We assess the performance of our models using two primary metrics for prediction intervals: (1) accuracy, representing the confidence in our PI estimates as determined by PICP, and (2) dimension, reflecting the resolution and quantified by PIW. We aim for a high PICP with a narrow PIW to ensure high-quality PIs.

We generate PIs with various NN-based PI estimation methodologies. First, we build a generalized model using Loss_S_ and then compare the findings of the generalized model with those of the models trained using the Loss_L_ and bootstrap approaches. The model using Loss_S_ demonstrates superior performance compared to the models using Loss_L_ and bootstrap, leading to reductions in PIW values of 22.4%, 7.9%, 16.7%, and 9.1% compared to the PIs generated by Loss_L_, and 19.3%, 21.1%, 23.6%, and 26.9% compared to the PIs generated by bootstrap, across PICP values of 50%, 75%, 85%, and 95%, respectively. The findings indicate that Loss_S_ outperforms Loss_L_ and bootstrap. Additionally, the results show a tradeoff between accuracy and dimension, whereby higher accuracy leads to a coarser dimension.

In the first case, we create a single model using all EDA signals from all subjects (population), which provides a generic model with reasonable performance and is valuable and applicable for pain intensity estimation in clinical settings. We then assess Loss_S_ performance on two other model-building approaches beyond the generalized model-building approach. In the second case, we develop personalized models for individual subjects, though the training data is very limited for each subject. Personalized models are not generalizable to new subjects. Since a new model may need to be created for each new subject or patient, it is not practical in clinical settings. In the third case, we develop a cluster-based hybrid approach, where individuals are grouped based on the similarity of their EDA features, and a dedicated model is created for each cluster of subjects. This approach provides the highest quality PIs with improved accuracies and lower dimensions, with average prediction interval widths of 0.44, 1.52, 1.86, and 2.5 for the 50%, 75%, 85%, and 95% prediction interval coverage probabilities, respectively. Importantly, this approach is practical in clinical settings because the same NN-based PI model can be used for a new patient, eliminating the need to construct a unique model for each individual. In conclusion, the NN-based PI algorithm with Loss_S_ effectively covers prediction uncertainty in pain intensity estimation. To our knowledge, this is the first study that estimated prediction intervals for pain intensity.

Data scarcity is a significant limitation in this study, particularly when developing personalized models. In future work, we will consider incorporating EMG, EEG, and video signals from individuals in the BioVid Heat Pain dataset and training models using these additional data sources. Genetic algorithms for PI estimation are computationally intense, primarily due to their iterative nature. This computational demand can become more pronounced when dealing with large datasets or high-dimensional optimization problems. Therefore, to overcome these challenges and enhance the efficiency of the optimization process, we will explore various optimization approaches, including particle swarm optimization, simulated annealing, and hybrid evolutionary algorithms.

In addition to its application in pain intensity estimation, the uncertainty quantification approach presented in this study holds promise for various other medical applications, including glucose level monitoring, blood pressure measurement, cardiovascular risk prediction, and drug dosage optimization. In each application, accurate and well-calibrated prediction intervals can significantly enhance the quality and reliability of medical decision-making, help manage patient expectations, and tailor interventions to individual patient needs.
